# Simultaneous TEP Inguinal Hernia Repair and Laparoscopic Cholecystectomy: A Retrospective Analysis of Safety, Cost-Effectiveness, and Outcomes

**DOI:** 10.3390/medicina62020330

**Published:** 2026-02-06

**Authors:** Zekai Serhan Derici, Berke Manoğlu, Tayfun Bişgin, Cihan Ağalar, Mert Kazancı, Tufan Egeli, Süleyman Özkan Aksoy

**Affiliations:** Department of Surgery, Faculty of Medicine, Dokuz Eylul University, 35340 Balcova, Turkey; berkemanoglu@hotmail.com (B.M.); tayfun.bisgin@deu.edu.tr (T.B.); cihanagalar@hotmail.com (C.A.); drmertkazanci@gmail.com (M.K.); tufanegeli@gmail.com (T.E.); suleyman.aksoy@yahoo.com (S.Ö.A.)

**Keywords:** TEP, inguinal hernia, laparoscopic cholecystectomy, simultaneous surgery, mesh infection, cost-effectiveness

## Abstract

*Background and Objectives*: The concurrent management of cholelithiasis and inguinal hernia remains a subject of surgical debate, primarily due to concerns regarding prosthetic mesh infection in a clean-contaminated field. This study evaluates the safety, cost-effectiveness, and functional outcomes of simultaneous totally extraperitoneal (TEP) repair and laparoscopic cholecystectomy (LC). *Materials and Methods:* A retrospective analysis was conducted on patients treated between 2015 and 2025 using a prospectively maintained institutional registry. The cohort was stratified into two arms: the Simultaneous Group (*n* = 16), undergoing synchronous TEP and LC; and the Staged Group (*n* = 13), managed via separate sessions. A strict “hernia-first” operative sequence was enforced to maintain sterility. Key endpoints included perioperative morbidity, long-term recurrence (mean follow-up: 53.9 months), economic burden, and quality of life (EuraHS-QoL). *Results:* No surgical site or prosthetic infections were documented in either cohort. The Simultaneous arm demonstrated a significantly reduced total operative duration compared to the cumulative time of the Staged approach (164.6 ± 44.9 vs. 226.2 ± 57.4 min; *p* = 0.003) and yielded a shorter hospitalization period (1.44 ± 0.51 vs. 2.31 ± 0.85 days; *p* = 0.002). Workforce reintegration was markedly accelerated in the simultaneous group (9.43 ± 3.36 vs. 24.69 ± 12.35 days; *p* < 0.001), translating to a total cost reduction of approximately 51% for unilateral cases. Conclusions: Concomitant TEP and LC represents a clinically viable and financially prudent strategy that does not compromise patient safety or prosthetic durability. Adherence to a strict “hernia-first” surgical sequence appears critical to preventing infectious morbidity. Given the superior resource utilization, this dual approach merits consideration as a primary therapeutic algorithm.

## 1. Introduction

Cholecystectomy and inguinal hernia repair constitute the cornerstones of general surgery practice globally. With an estimated 20 million inguinal hernia repairs performed annually worldwide, the surgical burden is substantial [1]. In the United States alone, over 800,000 inguinal hernia repairs and 750,000 cholecystectomies are undertaken each year [1,2]. Given that the prevalence of both conditions increases with age, their coexistence is a frequent clinical scenario. Treating these pathologies in separate sessions necessitates two distinct hospitalizations, repeated exposure to anesthesia, and a prolonged cumulative recovery period, all of which contribute to increased healthcare costs and workforce loss [3,4].

Minimally invasive interventions have established themselves as the primary therapeutic modality for both conditions. Specifically, the endoscopic totally extraperitoneal (TEP) approach is increasingly preferred for inguinal hernia repair due to its demonstrated clinical outcomes [5]. Similarly, laparoscopic cholecystectomy (LC) remains the gold standard for the management of symptomatic cholelithiasis [2].

Simultaneous TEP and LC offer clear theoretical advantages regarding patient convenience and healthcare resource utilization. However, widespread adoption of this combined approach has been limited by concerns regarding surgical site infection (SSI). TEP is a “clean” procedure involving the implantation of a prosthetic mesh, where infection can lead to severe morbidity necessitating explantation [6,7]. Conversely, LC is classified as a “clean-contaminated” procedure, as bile may harbor pathogens such as *Escherichia coli* and *Klebsiella pneumoniae* [8,9,10,11]. The potential for bacterial translocation or cross-contamination during a simultaneous operation remains a significant deterrent for many surgeons.

The primary objective of this research was to assess the clinical viability, safety profile, and cost-effectiveness of concomitant TEP inguinal hernia repair and laparoscopic cholecystectomy, with a specific focus on infectious morbidity and postoperative quality of life.

## 2. Materials and Methods

### 2.1. Study Design and Patient Selection

This retrospective cohort study was conducted using a prospectively maintained clinical database at the Department of General Surgery, Dokuz Eylul University Faculty of Medicine. The study protocol adhered to the ethical guidelines of the Declaration of Helsinki and received formal approval from the Institutional Review Board (Approval No: 2025/15-09 Date: 7 May 2025).

The study population comprised adult patients who underwent inguinal hernia repair between 2015 and 2025. Inclusion criteria were as follows: (1) Adult patients (>18 years); (2) Diagnosis of symptomatic cholelithiasis and concurrent inguinal hernia; and (3) American Society of Anesthesiologists (ASA) physical status I–III. Exclusion criteria were as follows: (1) Emergency surgery presentations (e.g., acute cholecystitis with sepsis, incarcerated or strangulated hernia); and (2) History of prior lower abdominal surgery that would preclude TEP access. Patients treated with both totally extraperitoneal (TEP) inguinal hernia repair and laparoscopic cholecystectomy (LC) were identified and stratified into two groups based on the surgical timing:

- Simultaneous Group (*n* = 16): Patients who underwent concomitant TEP and LC in a single anesthetic session.
- Staged Group (*n* = 13): Patients who underwent TEP and LC in two distinct surgical sessions.

### 2.2. Data Collection and Outcomes

Demographic and clinical data, including age, gender, body mass index (BMI), comorbidities (diabetes mellitus, coronary artery disease, hypertension, pulmonary disorders, and thyroid dysfunction), and surgical history were recorded prospectively.

The primary outcomes of the study were the incidence of surgical site infection (SSI), specifically mesh infection, and perioperative morbidity (classified according to the Clavien–Dindo system). The secondary outcomes included total operative time, length of hospital stay (LOS), time to return to daily activities, workforce reintegration time, hernia recurrence, total surgical costs, and postoperative quality of life (assessed via the EuraHS-QoL instrument).

### 2.3. Quality of Life Assessment

Postoperative functional outcomes were evaluated using the European Registry of Abdominal Wall Hernias Quality of Life (EuraHS-QoL) instrument. This survey was administered to all patients to assess pain, restriction of activities, and cosmetic satisfaction. For patients in the Staged Group, the EuraHS-QoL survey was administered separately after each surgical session (post-TEP and post-LC).

### 2.4. Surgical Technique

All procedures were performed under general anesthesia with patients in the supine position. A single dose of prophylactic antibiotic (2 g Cefazolin) was administered at induction.

**Figure 1:**
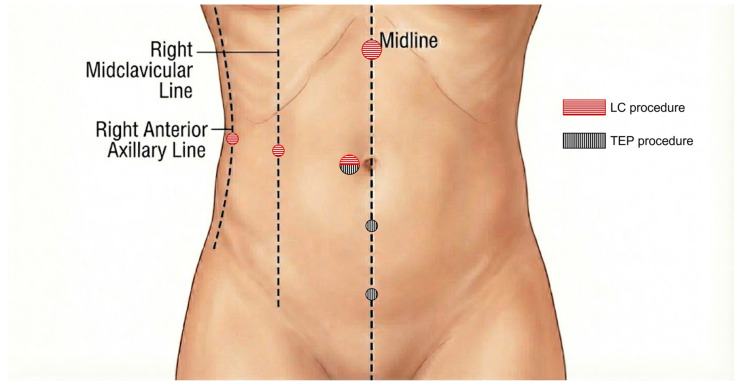
Surgical setup for simultaneous totally extraperitoneal (TEP) inguinal hernia repair and laparoscopic cholecystectomy. Schematic illustration of the trocar placement demonstrating the port sites for both TEP and LC.

- TEP Inguinal Hernia Repair: The preperitoneal space was accessed using a 10 mm balloon trocar at the infraumbilical margin. Two 5 mm working ports were placed in the midline between the pubic symphysis and the umbilicus. Following dissection, a polypropylene mesh (standard or self-fixating) was placed. In the event of peritoneal tearing, defects were closed using the LigaSure™ Vessel Sealing System (Medtronic, Minneapolis, MN, USA) and 5 mm endoscopic clips (LIGAMAX™ 5 Endoscopic Multiple Clip Applier-Ethicon Endo-Surgery, Inc., Cincinnati, OH, USA) to restore peritoneal integrity.
- Laparoscopic Cholecystectomy (LC): For isolated LC, pneumoperitoneum was established via a Veress needle. Access utilized a 10 mm infraumbilical optical trocar, a 10 mm subxiphoid trocar, and two 5 mm right upper quadrant working ports. The specimen was retrieved via the epigastric port using an endobag.
- Simultaneous Procedure Protocol: In the Simultaneous Group, the surgical protocol strictly adhered to a “clean-to-contaminated sequence” (hereinafter referred to as the “hernia-first approach”). This strategy mandates the completion of the aseptic TEP inguinal hernia repair prior to the commencement of the clean-contaminated laparoscopic cholecystectomy, thereby preserving the sterility of the preperitoneal mesh. Upon completion of the hernia repair and closure of the preperitoneal space, the procedure transitioned to cholecystectomy. A 10 mm trocar was inserted into the abdominal cavity at the periumbilical site using an open technique. Crucially, the TEP repair site was inspected from the intra-abdominal aspect to verify peritoneal integrity. Following confirmation that no peritoneal defects were present, standard LC ports were placed, and the cholecystectomy was completed. The schematic representation of the trocar placement and the postoperative view of a patient are presented in Figure 1.

### 2.5. Statistical Analysis

Statistical analysis was performed using IBM SPSS Statistics for Windows, Version 30.0 (IBM Corp., Armonk, NY, USA). Continuous variables were assessed for normality using the Shapiro–Wilk test and visual inspection of histograms. Normally distributed data are presented as mean ± standard deviation (SD), while non-normally distributed data are expressed as median (interquartile range). Categorical variables are reported as frequencies and percentages.

Quantitative variables were compared between independent groups using the Independent Samples *t*-test or the Mann–Whitney U test, depending on data distribution. Homogeneity of variance was assessed using Levene’s test; where the assumption of equal variances was violated, Welch’s *t*-test was utilized. For the Staged Group, which provided repeated measures (post-TEP and post-LC), within-group comparisons were performed using the Paired Samples *t*-test or the Wilcoxon Signed-Rank test. Categorical variables were compared using the Chi-square test or Fisher’s exact test. A *p*-value of <0.05 was considered statistically significant.

## 3. Results

### 3.1. Demographic and Clinical Characteristics

A total of 29 patients were included in the comparative analysis: 16 in the Simultaneous Group and 13 in the Staged Group. The overall cohort was predominantly male (27 males, 2 females), with a mean age of 59.62 ± 11.70 years and a mean BMI of 25.72 ± 3.59 kg/m^2^.

Baseline demographic characteristics and comorbidity profiles are summarized in Table 1. Statistical analysis revealed no significant differences between the groups regarding age, gender, BMI, or the prevalence of comorbidities such as diabetes, hypertension, and coronary artery disease (*p* > 0.05). Similarly, surgical history—including prior abdominal surgeries, cholecystitis attacks, and history of ERCP—was comparable between the two arms. Four patients underwent surgery for recurrent hernia. Polypropylene mesh was utilized in the majority of cases (ProGrip™ self-fixating mesh in 3 cases), with no significant divergence in mesh type selection.

### 3.2. Perioperative Outcomes and Safety

Perioperative data are detailed in Table 2. The Simultaneous Group demonstrated a significantly shorter total procedural duration (164.6 ± 44.9 min) compared to the cumulative operative time of the Staged Group (226.2 ± 57.4 min; *p* = 0.003). This substantial time difference in the Staged Group is attributable to the necessity of performing anesthesia induction, patient positioning, and surgical site preparation separately for each surgical session. Furthermore, the length of hospital stay (LOS) was significantly reduced in the Simultaneous Group compared to the cumulative LOS of the Staged Group (1.44 ± 0.51 days vs. 2.31 ± 0.85 days; *p* = 0.002).

Intraoperative complications were limited to minor peritoneal tearing (<3 cm), which occurred in three patients in the Simultaneous Group during TEP dissection. These defects were successfully repaired intraoperatively using clips and energy devices, and the procedures were completed endoscopically without conversion to open surgery.

No surgical site infections (SSI) or mesh infections were observed in either group during the perioperative period or the follow-up. Postoperative complications were classified according to the Clavien–Dindo system:

- Simultaneous Group: One patient developed urinary retention (Grade IIIa), and one patient required management for a hypertensive attack (Grade II).
- Staged Group: One patient experienced a hypertensive attack (Grade II), and one patient required treatment for bradycardia (Grade II).

Throughout the long-term surveillance period (mean 53.9 months), no instances of hernia recurrence were documented in any patient.

### 3.3. Economic Analysis and Functional Recovery

Economic outcomes are presented in Table 3. The staged approach resulted in a substantial increase in total surgical costs—approximately 51.44% higher for unilateral hernias and 34.86% higher for bilateral hernias compared to the simultaneous procedure.

Functional recovery was markedly expedited in the Simultaneous Group. The time to return to daily activities was 5.79 ± 2.69 days in the Simultaneous Group versus 12.54 ± 6.94 days in the Staged Group (*p* = 0.001). Furthermore, workforce reintegration was significantly faster in the Simultaneous Group (9.43 ± 3.36 days) compared to the Staged Group (24.69 ± 12.35 days; *p* < 0.001), representing an approximate 62% reduction in workforce loss.

### 3.4. Quality of Life Outcomes (EuraHS-QoL)

Postoperative quality of life outcomes, assessed via the EuraHS-QoL survey, are presented in Table 4. To provide a granular analysis, the outcomes of the Simultaneous Group were compared against the distinct surgical phases (TEP phase and LC phase) of the Staged Group.

Analysis of QoL outcomes revealed comparable pain profiles across all comparisons. No significant differences were observed in resting pain, daily activity pain, or exercise pain between the Simultaneous group and either phase of the Staged group (*p* > 0.05). Furthermore, within the Staged group, no significant deterioration in QoL scores was detected between the first (TEP) and second (LC) procedures (Table 4). Patient satisfaction was high in the Simultaneous Group, with mean cosmetic and general satisfaction scores of 9.43 and 9.71, respectively. Notably, 100% of patients in the Simultaneous Group stated they would choose the combined method again if given the choice.

## 4. Discussion

The simultaneous management of coexisting intra-abdominal pathologies represents a significant advancement in minimally invasive surgery, aiming to optimize patient outcomes while reducing healthcare burdens. The results of this investigation confirm that the simultaneous execution of totally extraperitoneal (TEP) inguinal hernia repair and laparoscopic cholecystectomy (LC) is a clinically safe, feasible, and economically advantageous approach that preserves postoperative quality of life.

The primary concern limiting the widespread adoption of this combined approach has historically been the risk of prosthetic mesh infection, given that LC is classified as a clean-contaminated procedure [12]. This concern is grounded in robust bacteriological evidence. While earlier studies such as Sattar et al. reported positive bile cultures in 36% of patients, contemporary data indicate significantly higher contamination rates [13]. A recent comprehensive analysis of 9939 cases revealed a culture positivity rate of 51.85%, and Gandhi et al. reported a positivity rate of 46% in a tertiary care setting [8,14]. Furthermore, standard cultures may underestimate the true bacterial load; Matyjas et al. questioned whether cholelithiasis is ‘always infected’, identifying bacterial DNA in gallstones even in asymptomatic cases [15]. Similarly, recent metagenomic analyses have unveiled complex microbial communities within the biliary tract even in culture-negative patients [9].

Despite this substantial risk of bacterial translocation and occult contamination, our study observed zero instances of mesh infection or surgical site infection (SSI) in the simultaneous cohort. These findings align with a recent systematic review by Doluweera et al., which analyzed 199 patients and reported no cases of mesh infection [16]. Similarly, Praveen Raj et al. reported an infection rate of only 0.8% in a large series, concluding that concomitant clean-contaminated surgeries do not significantly alter infective morbidity [17]. Our results corroborate the consensus that with appropriate prophylactic antibiotics and meticulous technique, the “clean-contaminated” nature of LC does not contraindicate simultaneous mesh placement [18].

Simultaneous laparoscopic surgery has gained acceptance not only in elective cases but also in emergency settings. A recent study by Pogorelić et al. demonstrated that a single-stage laparoscopic approach for acute appendicitis and inguinal hernia is both safe and feasible, with no increase in complication rates compared to staged procedures [19]. Our findings in the elective setting (cholecystectomy and TEP repair) are consistent with this growing body of evidence supporting the safety of concurrent laparoscopic interventions.

A critical determinant of safety in our protocol is the “hernia-first” surgical sequence. We strictly adhered to performing TEP (clean) prior to LC (clean-contaminated). This sequence maintains the integrity of the peritoneum during hernia repair, isolating the preperitoneal mesh from the intraperitoneal cavity before any potential exposure to bile or bacteria occurs [20]. Furthermore, this “hernia-first” order eliminates the risk of cross-contamination of surgical instruments, allowing the same instrument set to be used for both procedures. Conversely, if LC were performed first, an accidental gallbladder perforation would create significant pressure to avoid peritoneal breaches during the subsequent TEP, making the procedure technically more stressful and risky [4,12,21]. By prioritizing TEP, the mesh is securely implanted and the preperitoneal space is closed before the cholecystectomy commences.

From a health economics perspective, our findings highlight the substantial efficiency of the single-session approach. The simultaneous group demonstrated significantly shorter cumulative operative times compared to the staged group. This temporal advantage is primarily attributable to the single-session consolidation, which eliminates the need to repeat anesthesia induction, patient positioning, and surgical site preparation. In parallel with this intraoperative efficiency, the simultaneous approach also yielded a significantly reduced total length of hospital stay. Unlike the staged protocol, which mandates two distinct admissions and recovery periods, the combined procedure requires only a single convalescence phase, thereby preventing the logistical doubling of hospitalization days. While early studies suggested higher costs for combined procedures, contemporary data support our findings of economic efficiency [22]. Claus et al. reported that the staged approach was 43% more expensive than the combined procedure [20]. Our analysis indicated a total cost reduction of approximately 51% for unilateral cases, driven by the elimination of a second hospital admission and the optimized use of disposable equipment.

Postoperative recovery and quality of life (QoL) are paramount in elective surgery. Workforce reintegration was significantly accelerated in the Simultaneous Group (9.43 days) compared to the Staged Group (24.69 days), effectively reducing labor productivity loss by more than 50%. This mirrors findings by Tsimoyiannis et al. regarding rapid return to normal activity [23]. Uniquely, we utilized the EuraHS-QoL instrument to objectively assess functional outcomes, revealing that pain levels and physical restriction scores in simultaneous patients were statistically indistinguishable from those undergoing isolated procedures. This confirms that the combined approach does not impose an additional burden on the patient’s postoperative comfort [24].

To contextualize our findings, we conducted a review of the literature comparing simultaneous inguinal hernia repair and LC (Table 5). Analysis of studies spanning from 1994 to 2023 reveals a consistent safety profile across various techniques (TAPP, TEP, IPOM) [3,4,17,20,21,22,23,24,25,26,27,28,29]. Our series is distinguished by its extended follow-up period (mean 53.9 months), providing compelling validation regarding the long-term integrity of the repair and the absence of delayed mesh-related adverse events. The evolution of clinical consensus, detailed in Table 6, increasingly underscores the fiscal and logistical advantages of this strategy [3,29].

### Limitations

The interpretation of our findings warrants consideration of certain limitations. Primarily, the retrospective, monocentric design and the non-randomized allocation of patients may introduce selection bias. The allocation to simultaneous versus staged surgery was largely determined by patient preference and logistical operating room availability, rather than a randomized protocol. Secondly, while the sample size was sufficient to demonstrate feasibility, the study was not statistically powered to detect exceedingly rare complications, specifically mesh infection, which would require a significantly larger cohort to establish incidence rates with high confidence. Finally, as this study was conducted in a high-volume tertiary center by a dedicated laparoscopic team, the safety profile and operative efficiency observed may not be fully generalizable to settings with limited experience in totally extraperitoneal (TEP) repair.

## 5. Conclusions

In conclusion, the concomitant execution of TEP inguinal hernia repair and laparoscopic cholecystectomy represents a clinically sound strategy that combines fiscal efficiency with a robust safety profile. Our findings indicate that this dual approach expedites recovery and workforce reintegration without elevating the risk of septic morbidity or hernia recurrence. Consequently, this combined intervention merits consideration as a primary management algorithm for eligible candidates, provided that the “hernia-first” surgical sequence is strictly observed to maintain sterility. Validation of these promising outcomes through future prospective, multi-institutional investigations would further solidify its place in general surgical practice.

## Figures and Tables

**Table 1:** Baseline demographic and clinical characteristics of the patients.

Characteristic	Simultaneous Group (*n* = 16)	Staged Group (*n* = 13)	*p* Value
Age (years), mean ± SD	57.88 ± 13.75	61.77 ± 8.61	0.361
Gender, *n* (%)			0.488
Male	14 (87.5)	13 (100)	
Female	2 (12.5)	0 (0)	
BMI (kg/m^2^), mean ± SD	26.05 ± 4.06	25.32 ± 3.02	0.612
Weight (kg), mean ± SD	77.73 ± 12.10	76.17 ± 9.42	0.716
Height (cm), mean ± SD	172.87 ± 5.65	173.50 ± 5.98	0.780
Comorbidities, *n* (%)			
Hypertension	8 (50.0)	4 (30.8)	0.451
Diabetes Mellitus	3 (18.8)	2 (15.4)	1.000
Coronary Artery Disease	3 (18.8)	5 (38.5)	0.406
Pulmonary Disease	2 (12.5)	0 (0)	0.488
Thyroid Disease	1 (6.3)	3 (23.1)	0.299

SD = Standard Deviation. BMI = Body Mass Index.

**Table 2:** Perioperative outcomes and complications.

Variable	Simultaneous Group (*n* = 16)	Staged Group (*n* = 13)	*p* Value
Operative time (min), mean ± SD	164.63 ± 44.91	226.23 ± 57.43	0.003
Length of hospital stay (days), mean ± SD	1.44 ± 0.51	2.31 ± 0.85	0.002
Peritoneal tearing, *n* (%)	3 (18.8)	0 (0)	0.232
Conversion to open surgery, *n* (%)	0 (0)	0 (0)	—
Surgical Site Infection, *n* (%)	0 (0)	0 (0)	—
Postoperative complications, *n* (%)	2 (12.5)	2 (15.4)	0.558

SD = Standard Deviation.

**Table 3:** Cost comparison analysis between simultaneous and staged procedures.

Surgical Strategy	Total Cost (USD)	Additional Cost Burden
Unilateral Inguinal Hernia + LC		
Simultaneous (Single Session)	2001.54	—
Staged (Separate Sessions)	3031.22	+51.44%
Bilateral Inguinal Hernia + LC		
Simultaneous (Single Session)	2952.98	—
Staged (Separate Sessions)	3982.66	+34.86%

LC = Laparoscopic Cholecystectomy.

**Table 4:** Comparison of postoperative quality of life scores (EuraHS-QoL) among surgical groups.

	Simultaneous Group (*n* = 16)	Staged Group (*n* = 13)		*p* Value (Sim vs. Post-TEP) ^a^	*p* Value (Sim vs. Post-LC) ^a^	*p* Value (Post-TEP vs. Post-LC) ^b^
Quality of Life Parameter	TEP + LC	Post TEP Phase	Post LC Phase			
Pain Scores (VAS), mean ± SD						
Resting pain	1.93 ± 1.64	2.00 ± 1.96	2.23 ± 2.49	0.782	0.711	0.756
Pain during daily activities	2.14 ± 1.83	2.62 ± 2.43	2.31 ± 1.89	0.640	0.820	0.487
Pain during exercise	2.93 ± 2.20	2.92 ± 3.10	2.62 ± 2.73	0.857	0.744	0.367
Physical Restriction, mean ± SD						
Restriction in daily activities	2.14 ± 2.14	1.85 ± 2.27	1.62 ± 1.94	0.562	0.508	0.190
Restriction in sports/exercise	3.64 ± 2.41	4.23 ± 3.77	4.46 ± 3.78	0.473	0.505	0.190
Patient Satisfaction, mean ± SD						
Cosmetic satisfaction	9.43 ± 0.85	9.38 ± 1.45	9.00 ± 2.04	0.637	0.477	0.175
General result satisfaction	9.71 ± 0.61	9.62 ± 0.65	9.54 ± 0.66	0.516	0.479	0.337

Data presented as Mean ± Standard Deviation. SD = Standard Deviation. VAS = Visual Analog Scale (0–10). ^a^ Calculated using Independent Samples *t*-test. (For variables with unequal variances, Welch’s *t*-test values are reported.) ^b^ Calculated using Paired Samples *t*-test.

**Table 5:** Review of the literature comparing simultaneous inguinal hernia repair and laparoscopic cholecystectomy.

Author (Year)	Study Design	*n*	Hernia Repair	Antibiotic Prophylaxis	First Proced.	Op.Time (Min)	Mesh Type	Drain Usage (%)	Mesh Infection (*n*)	Local Complic. (*n*)	LOS (Days)	Follow-Up (Months)	Recurrence (%)
Tsimoyiannis et al. (1994) [23]	Retrosp.	6	IPOM	3rd Gen. Cephalosporin	LC	103 (90–120)	ePTFE	16.7	0	0	1	11 (8–13)	0
Sarli et al. (2001) [22]	RCT	30	TAPP	Yes	HR	121 ± 32	PP	100	0	Hematoma (2), Hydrocele (1)	2.2 ± 1	36	3.3
Al-Dowais (2009) [26]	Case Rep.	1	TAPP	Yes	LC	110	PP	0	0	0	1	NR	0
Savita et al. (2010) [25]	Retrosp.	23	TEP/TAPP	NR	HR	NR	NR	NR	0	0	NR	NR	0
Suh et al. (2012) [21]	Case Rep.	1	TEP	NR	HR	98.9	PP	0	0	0	4	NR	0
Lehmann et al. (2014) [4]	Retrosp.	8	TAPP	Yes	HR	55 (30–60)	Mixed *	100	0	0	3.6 (2–7)	NR	0
Praveen Raj et al. (2015) [17]	Retrosp.	120	IPOM	3rd Gen. Cephalosporin	LC	136 (112–172)	Composite	NR	0	Seroma (1)	2.7 (1–5)	NR	0
Arafat et al. (2017) [27]	Case Rep.	2	TAPP	Cefotaxime	LC	NR	PP	100	0	0	1	3	0
Rabie et al. (2018) [28]	Case Rep.	1	TAPP	Amoxicillin/Clavulanic acid	LC	NR	NR	0	1	0	3	NR	0
Hayakawa et al. (2019) [3]	Retrosp.	17	TAPP	NR	HR	157 ± 39	NR	0	0	0	3.2 ± 0.6	3	0
Quezada et al. (2019) [24]	Retrosp.	21	TAPP	Cefazolin	LC: 13, HR: 8	111 (60–180)	Mixed **	0	0	0	NR	40 (4–89)	4.8
Claus et al. (2021) [20]	Retrosp.	46	TEP/TAPP	NR	HR	111 (60–210)	Heavyweight PP	0	0	0	1.1	47.1 (3–111)	2.2
Akay et al. (2023) [29]	Retrosp.	38	TAPP	Cefazolin	LC	124 ± 26	PP	100	0	0	2.1 ± 0.4	NR	2.6
Current Study (2025)	Retrosp.	29	TEP	Cefazolin	HR	164.6 ± 44.9	PP(26), ProGrip™ (3)	0	0	0	1.4 ± 0.5	53.9	0

Retrosp: Retrospective, Case Rep: Case Report, RCT = Randomized Controlled Trial, IPOM = Intraperitoneal Onlay Mesh, TAPP = Transabdominal Preperitoneal, TEP = Totally Extraperitoneal, LC = Laparoscopic Cholecystectomy, HR = Inguinal Hernia Repair, ePTFE = expanded Polytetrafluoroethylene, PP = Polypropylene, LOS = Length of Stay, NR = Not Reported. Values are presented as mean ± SD, median (range), or number unless otherwise indicated. * UltraPro, Bard 3D Mesh, Prolene. ** Polypropylene (12), Polyester (8), Polyvinylidene fluoride (1).

**Table 6:** Key conclusions of the included studies.

Author (Year)	Key Conclusion
Tsimoyiannis et al. (1994) [23]	Simultaneous repair is easy, well-tolerated, and cost-saving with no increase in morbidity.
Sarli et al. (2001) [22]	Totally laparoscopic approach improves early postoperative comfort compared to open repair but requires advanced expertise.
Al-Dowais (2009) [26]	Trans-umbilical (single incision) approach is feasible and safe without increased morbidity.
Savita et al. (2010) [25]	Provides single anesthesia benefit without adding to postoperative morbidity or hospital stay.
Suh et al. (2012) [21]	Single-incision synchronous TEP and cholecystectomy is feasible with favorable cosmetic results.
Lehmann et al. (2014) [4]	Feasible and safe procedure; does not prolong convalescence compared to separate procedures.
Praveen Raj et al. (2015) [17]	Safe in selected cases; mesh infection rate (0.8%) is comparable to isolated IPOM repair.
Arafat et al. (2017) [27]	Safe in selected cases; prophylactic antibiotics effectively minimize surgical site infection risk.
Rabie et al. (2018) [28]	Feasible and safe even in the setting of incarcerated femoral hernia.
Hayakawa et al. (2019) [3]	Safe standard procedure; significantly reduces economic burden and hospital stay compared to two separate hospitalizations.
Quezada et al. (2019) [24]	Safe and effective; associated with excellent postoperative quality of life and no mesh infections.
Claus et al. (2021) [20]	Safe with no mesh infection increase; reduces hospital costs by ~30% and increases patient satisfaction.
Akay et al. (2023) [29]	Safe to perform; performing cholecystectomy first did not increase mesh infection rates.
Current Study (2025)	Superior cost–benefit, earlier return to work, high QoL.

TEP = Totally Extraperitoneal, IPOM = Intraperitoneal Onlay Mesh, QoL = Quality of life scores.

## Data Availability

The data sets used and/or analyzed during the current study are available from the corresponding author on reasonable request.

## Abbreviations

The following abbreviations are used in this manuscript:

TEP	Totally Extraperitoneal
LC	Laparoscopic Cholecystectomy
EuraHS-QoL	European Registry of Abdominal Wall Hernias Quality of Life
QoL	Quality of Life
BMI	Body Mass Index
ASA	American Society of Anesthesiologists
DM	Diabetes Mellitus
CAD	Coronary Artery Disease
HT	Hypertension
ERCP	Endoscopic Retrograde Cholangiopancreatography
LOS	Length of Hospital Stay
SD	Standard Deviation
SSI	Surgical Site Infection
TAPP	Transabdominal Preperitoneal
IPOM	Intraperitoneal Onlay Mesh

## References

[1] Francesco F., Mola D., Huerta S., Garza A.M. (2025). A Systematic Review of Open, Laparoscopic, and Robotic Inguinal Hernia Repair: Management of Inguinal Hernias in the 21st Century. J. Clin. Med..

[2] Alberton A., Peltz E.D. (2024). Cholecystectomy. Surg. Clin. N. Am..

[3] Hayakawa S., Hayakawa T., Inukai K., Miyai H., Yamamoto M., Kitagami H., Shimizu Y., Tanaka M. (2019). Simultaneous Transabdominal Preperitoneal Hernia Repair and Laparoscopic Cholecystectomy: A Report of 17 Cases. Asian J. Endosc. Surg..

[4] Lehmann A., Piatkowski J., Nowak M., Jackowski M., Pawlak M., Witzling M., Śmietanski M. (2014). Simultaneous TAPP (Transabdominal Pre-Peritoneal Technique) for Inguinal Hernia and Cholecystectomy—A Feasible and Safe Procedure. Pol. Prz. Chir. Pol. J. Surg..

[5] Simons M.P., Smietanski M., Bonjer H.J., Bittner R., Miserez M., Aufenacker T.J., Fitzgibbons R.J., Chowbey P.K., Tran H.M., Sani R. (2018). International Guidelines for Groin Hernia Management. Hernia.

[6] Mavros M.N., Athanasiou S., Alexiou V.G., Mitsikostas P.K., Peppas G., Falagas M.E. (2011). Risk Factors for Mesh-Related Infections after Hernia Repair Surgery: A Meta-Analysis of Cohort Studies. World J. Surg..

[7] Ambrose N.P., Stephen D.P.T., DK T., Samuel B.R., Rebekah G., Chase S. (2026). Staged Approach to Chronic Mesh Infection Following Hernia Repair: A Single-Center Experience. Hernia.

[8] Zheng X., Yan Y., Li X., Liu M., Zhao X., He J., Zhuang X. (2024). Microbial Characteristics of Bile in Gallstone Patients: A Comprehensive Analysis of 9,939 Cases. Front. Microbiol..

[9] Park W., Park J. (2024). A Comparative Investigation of the Bile Microbiome in Patients with Choledocholithiasis and Cholecystolithiasis through Metagenomic Analysis. Int. J. Mol. Sci..

[10] Liu Y., Yao B., Yang X., Sun Q., Yang X., Liang L. (2025). Research Progress on the Role of Microbiota in the Pathogenesis of Gallstone Disease. Front. Microbiol..

[11] He L., Wang X., Fan G., Zhao Y. (2022). Hernia Mesh Infection Treatment Following the Repair of Abdominal Wall Hernias: A Single-Center Experience. Front. Surg..

[12] Peponis T., Eskesen T.G., Mesar T., Saillant N., Kaafarani H.M.A., Yeh D.D., Fagenholz P.J., de Moya M.A., King D.R., Velmahos G.C. (2018). Bile Spillage as a Risk Factor for Surgical Site Infection after Laparoscopic Cholecystectomy: A Prospective Study of 1,001 Patients. J. Am. Coll. Surg..

[13] Sattar I., Aziz A., Rasul S., Mehmood Z., Khan A. (2017). Frequency of Infection in Cholelithiasis. J. Coll. Physicians Surg. Pak..

[14] Gandhi H., Bhargava G.S., Bansal D., Singh K. (2020). Morphological Spectrum of Gallstone and Bacteriology of Bile in Patient of Cholelithiasis Visiting Tertiary Care Centre in North India. Int. Surg. J..

[15] Matyjas T., Pomorski L., Witas H., Płoszaj T., Matyjas K., Kaczka K. (2017). Cholelithiasis—Always Infected?. Pol. J. Surg..

[16] Doluweera D., Silva O., Seneviratne S.L., De Zoysa I. (2025). Safety of Simultaneous Laparoscopic Cholecystectomy and Inguinal Hernia Repair: A Systematic Review. J. Laparoendosc. Adv. Surg. Tech..

[17] Raj P.P., Ganesh M.K., Senthilnathan P., Parthasarathi R., Rajapandian S., Palanivelu C. (2015). Concomitant Laparoscopic Intraperitoneal Onlay Mesh Repair with Other Clean Contaminated Procedures—Study of Feasibility and Safety. J. Laparoendosc. Adv. Surg. Tech..

[18] Birolini C., de Miranda J.S., Tanaka E.Y., Utiyama E.M., Rasslan S., Birolini D. (2020). The Use of Synthetic Mesh in Contaminated and Infected Abdominal Wall Repairs: Challenging the Dogma-A Long-Term Prospective Clinical Trial. Hernia.

[19] Pogorelić Z., Ødeverp A., Jukić M. (2025). The Safety and Feasibility of Single-Stage Versus Staged Laparoscopic Approach for Acute Appendicitis with Inguinal Hernia in Pediatric Patients: A Comparative Study. J. Clin. Med..

[20] Claus C.M.P., Ruggeri J.R.B., Ramos E.B., Costa M.A.R., Andriguetto L., de Freitas A.C.T., Coelho J.C.U. (2021). Simultaneous laparoscopic inguinal hernia repair and cholecystectomy: Does it cause mesh infection?. Arq. Bras. Cir. Dig..

[21] Suh H.-H., Cho Y.K., Rheu H.G. (2012). Synchronous Cholecystectomy and Totally Extraperitoneal (TEP) Herniorrhaphy Using an Umbilical Incision. J. Minim. Invasive Surg..

[22] Sarli L., Villa F., Marchesi F. (2001). Hernioplasty and Simultaneous Laparoscopic Cholecystectomy: A Prospective Randomized Study of Open Tension-Free versus Laparoscopic Inguinal Hernia Repair. Surgery.

[23] Tsimoyiannis E.C., Paizis J.B., Siakas P., Lekkas E.T. (1994). Cholecystectomy and Hernioplasty During the Same Laparoscopic Procedure. Surg. Laparosc. Endosc..

[24] Quezada N., Maturana G., Pimentel E., Crovari F., Muñoz R., Jarufe N., Pimentel F. (2019). Simultaneous TAPP Inguinal Repair and Laparoscopic Cholecystectomy: Results of a Case Series. Hernia.

[25] Savita K.S., Khedkar I., Bhartia V.K. (2010). Combined Procedures with Laparoscopic Cholecystectomy. Indian J. Surg..

[26] Al-Dowais A. (2009). Total Trans-Umbilical Laparoscopic Cholecystectomy And Inguinal Hernia Repair. Internet J. Surg..

[27] Arafat S., Alsabek M.B. (2017). Simultaneous Laparoscopic Cholecystectomy and Transabdominal Preperitoneal Hernioplasty: Two Case Reports Evaluate the Safety and Surgical Complications. Clin. Case Rep..

[28] Rabie M.E., Hummadi A., Osama M. (2018). Synchronous Laparoscopic Cholecystectomy and Mesh Repair of Incarcerated Femoral Hernia: Is It Feasible?. Saudi Surg. J..

[29] Akay T., Çalta A.F. (2023). Is It Safe to Perform Laparoscopic Cholecystectomy and Transabdominal Preperitoneal Hernia Repair Simultaneously?. J. Gen. Med. Genel Tıp Derg..

